# Long duration anaesthesia in pigs with an infusion of alfaxalone and dexmedetomidine

**DOI:** 10.1002/vms3.953

**Published:** 2022-09-22

**Authors:** Irving Kat, Benjamin J. Ahern, Jayesh Dhanani, Grant Whitten, Nicholas Cowling, Wendy Goodwin

**Affiliations:** ^1^ School of Veterinary Science The University of Queensland Gatton Australia; ^2^ Department of Intensive Care Medicine Royal Brisbane and Women's Hospital Brisbane Australia; ^3^ University of Queensland Centre of Clinical Research, Faculty of Medicine, Level 8 The University of Queensland Herston Australia

**Keywords:** TIVA, pig, alfaxalone, dexmedetomidine

## Abstract

Pigs are commonly maintained on total intravenous anaesthesia when used in comparative medical research to study controlled manual ventilation of the lung. In this case study, four pigs were anaesthetised with a total intravenous anaesthetic infusion of alfaxalone and dexmedetomidine for up to 24 h whilst being mechanically ventilated. Cardiovascular parameters, blood gas values and body temperature were minimally affected throughout the anaesthetic period. Additional analgesia is recommended when utilising this drug combination for procedures that involve noxious stimuli.

## 1 INTRODUCTION

In respiratory research, the similarities between the pig and the human lung have made the pig a common model in comparative medical research studying ventilation and respiration (Judge et al., 2014). To facilitate research into critical care ventilators using pigs, intravenous infusions of anaesthetic drugs may be used to maintain pigs at an anaesthetic plane such that they can be intubated to allow mechanical ventilation. Alfaxalone is a neuroactive steroid that is commercially available in an unpreserved (Alfaxan®) and preserved (Alfaxan® Multidose) formulation that is registered for use in veterinary medicine as an intravenous induction and maintenance anaesthetic agent in dogs and cats. In pigs, alfaxalone has been used to induce and maintain anaesthesia and reported to have minimal cardiovascular effects at clinical does rates (Bigby et al., 2017; Lervik et al., 2020; Santos Gonzalez et al., 2013). As such, alfaxalone is an alternative choice as an intravenous maintenance anaesthetic agent in the pig, particularly if there is a potential shortage of commonly used human drugs (e.g., propofol) such as during the COVID‐19 pandemic. To the authors’ knowledge, the use of an intravenous infusion of alfaxalone and dexmedetomidine to maintain long duration anaesthesia (12–24 h) has not previously been reported in pigs.

## 2 CASE REPORT

In a recent pilot study, a combination of alfaxalone (Alfaxan® Multidose, Jurox Pty Ltd., Rutherford, NSW, Australia) and dexmedetomidine (Dexdomitor, Jurox Pty Ltd.) was used to maintain long duration anaesthesia (12–24 h) in pigs that were mechanically ventilated to assess a rapid prototyped mechanical ventilator (Dhanani et al., 2020). All procedures were performed with approval from the University of Queensland Animal Ethics Committee (SVS/142/20).

Four female Large White (*Sus scrofa domesticus*) pigs aged approximately 10 weeks and weighing 35–40 kg were premedicated with intramuscular (IM) ketamine 10 mg/kg (Ketamil, Mavlab Pty Ltd, Slacks Creek, QLD, Australia), dexmedetomidine 10 μg/kg and methadone 0.25–0.4 mg/kg (Methodyne, Jurox Pty Ltd.). Once recumbent, anaesthesia was induced with isoflurane and oxygen delivered via a face mask connected to a circle system. The trachea was intubated with a cuffed endotracheal tube and anaesthesia initially maintained with isoflurane and oxygen. The auricular vein was catheterised and compound sodium lactate (Hartmann's Solution, Baxter Healthcare Australia, NSW), an isotonic crystalloid intravenous fluid, was administered intravenously at 3–4 ml/kg/h with intermittent bolus doses (10 ml/kg) administered to treat hypotension (defined as mean arterial pressure of 60 mmHg and below). The auricular artery was also catheterised to measure arterial blood pressure and the urinary bladder catheterised to permit continuous drainage of urine.

An hour after induction of anaesthesia, prior to the ventilator trial beginning, pigs were positioned in sternal recumbency, and inhalant maintenance anaesthesia was changed to total intravenous anaesthesia (TIVA) with a variable rate intravenous infusion of alfaxalone and dexmedetomidine using syringe drivers (Alaris® GH Plus, Becton Dickinson, Hamilton, QLD, Australia). Once pigs were judged to be adequately anaesthetised, controlled manual ventilation (CMV) was commenced using either a test prototype ventilator or a commercial ventilator (Ulco Campbell Ventilator EV500, Ulco Engineering Pty Ltd, Marrickville, NSW, Australia) over a 12‐ to 24‐h period with an air and oxygen mixture. To test the functionality of the prototype ventilator, the ventilator settings were periodically changed. Further details regarding the ventilators are already described (Dhanani et al., 2020), and ventilator settings are reported in Table 2. Cardiorespiratory variables and clinical observations of anaesthetic depth were monitored continuously during the study using a multiparameter anaesthetic monitor (BM7vet, Bionet, Guro‐gu, Seoul, Republic of Korea), and values were recorded every 5–10 min. Variables monitored included heart rate, arterial blood pressure, pulse oximetry, electrocardiogram, body temperature, end tidal carbon dioxide, palpebral reflex, eye position and jaw tone. Additionally, arterial blood samples were collected anaerobically every 2 h to measure pH, PaO_2_, PaCO_2_ and HCO_3_‐ using a point‐of‐care blood gas analyser (GEM Premier 3500, Werfen, Artarmon, New South Wales, Australia). At the completion of the experimental period, pigs were euthanised with an overdose of pentobarbitone sodium (185 mg/kg IV) (Lethabarb, Virbac Animal Health, Milperra NSW, Australia).

TIVA was maintained for 12 h only in Pigs 1 and 2 due to failure of the test ventilator and for 24 h in Pigs 3 and 4. During the study period, TIVA with the variable rate infusion, reported as median (range), was alfaxalone 5.3 (2.9–5.7) mg/kg/h and dexmedetomidine 3.0 (1.0–5.3) μg/kg/h (Table 1). Additional drugs were administered as part of a multi‐modal approach to maintain a suitable depth of anaesthesia. In all instances prior to additional drug administration, pigs were judged to be too lightly anaesthetised, evidenced by increased jaw tone and swallowing or head movement in response to whole‐body manipulation for thoracic radiographs. Pig 3 received these doses of methadone (Methadone Ilium, Troy Animal Healthcare, Glendenning, New South Wales, Australia) 0.2 mg/kg intramuscularly at hours 5, 9, and 18 post‐induction, whilst Pig 4 received a single dose of 0.2 mg/kg intramuscularly at 8 h post‐induction). No shaking or muscle tremors were observed during the anaesthetised period. Cardiorespiratory variables at each blood gas sampling point are shown in Table 2.

**TABLE 1:** Variable intravenous infusion rates of alfaxalone and dexmedetomidine for four pigs maintained on total intravenous anaesthesia and mechanically ventilated over a period of 12–24 h

	Pig 1	Pig 2	Pig 3	Pig 4
Weight (kg)	40	38	35	35
Median (min‐max) alfaxalone infusion (mg/kg/h)	5	4.65 (4−5.3)	5.7 (2.9−5.7)	5.1 (4.3−5.7)
Median (min‐max) dexmedetomidine infusion (μg/kg/h)	4	3.15 (1.1−5.3)	3 (1.1−3.4)	3 (3−4)
Intramuscular methadone (mg)	‐	‐	21	21
Duration of TIVA (hours)	12	12	24	24

**TABLE 2:** Physiological Parameters at each blood gas sampling time point for four pigs maintained under total intravenous anaesthesia whilst mechanically ventilated

		Ventilator settings						Blood gas				Cardiovascular					Other	
Hour		PIP	RR	TV	FiO_2_	PEEP	I:E ratio	pH	PO_2_	PCO_2_	HCO_3_‐	HR	SAP	DAP	MAP	SPO_2_	ETCO_2_	Temp
0	Pig 1	17	19	‐	52	5	‐	7.51	340	44	35.1	110	121	81	95	99	43	37.7
	Pig 2	19	23	350	97	5	‐	7.47	508	49	35.7	100	129	81	100	95	52	38.1
	Pig 3	20	17	465	54	5	1:2	7.46	279	49	34.8	83	111	62	78	95	51	39
	Pig 4	22	20	425	60	5	‐	7.48	214	46	34.3	85	95	43	60	91	55	39.1
2	Pig 1	21	23	450	47	5	‐	7.52	229	39	31.8	107	112	67	81	99	42	38.4
	Pig 2	20	23	350	67	5	‐	7.49	305	44	33.5	107	127	78	94	98	50	39.1
	Pig 3	20	17	472	57	5	2:5	7.48	286	47	35	‐	127	91	105	97	48	38.4
	Pig 4	22	20	425	48	5	1:1.97	7.45	234	46	32	76	106	63	80	99	52	38.7
4	Pig 1	20	21	‐	54	10	‐	7.42	263	45	29.2	103	121	71	89	99	47	38.6
	Pig 2	20	23	350	54	5	‐	7.49	267	44	33.5	110	129	79	97	97	50	39.1
	Pig 3	19	16	471	57	5	‐	7.44	289	48	32.6	77	116	80	96	98	47	38
	Pig 4	22	20	430	47	5	1:1.97	7.46	232	47	33.4	83	116	76	94	99	53	38.8
6	Pig 1	20	21	‐	52	10	‐	7.37	271	49	27.9	106	102	60	73	99	48	38.7
	Pig 2	20	23	350	54	10	‐	7.46	267	48	34.1	101	122	74	92	96	51	38.3
	Pig 3	27	16	479	54	10	‐	7.45	268	48	33.4	79	109	69	84	98	50	38.7
	Pig 4	23	24	375	49	10	1:1.59	7.46	243	45	32	83	99	55	73	99	50	38.8
8	Pig 1	30	21	‐	52	10	‐	7.38	271	52	29.7	110	105	57	70	98	49	38.7
	Pig 2	20	23	350	54	10	‐	7.47	263	48	34.9	105	131	79	98	95	52	39.2
	Pig 3	27	20	472	50	10	‐	7.45	248	47	32.7	90	117	72	88	98	50	38.4
	Pig 4	24	22	375	49	10	1:1.82	7.46	253	45	32	79	105	58	76	99	48	38.6
10	Pig 1	‐	21	‐	53	‐	‐	‐	‐	‐	‐	140	108	57	71	98	60	39
	Pig 2	‐	‐	‐	‐	‐	‐	‐	‐	‐	‐	105	130	78	98	95	51	39.5
	Pig 3	34	22	‐	48	15	‐	7.39	235	52	31.5	126	112	74	87	98	54	38.8
	Pig 4	28	28	250	44	15	1:1.3	7.4	256	51	31.6	96	100	59	75	99	58	38.3
12	Pig 1	*	25	*	*	*	*	‐	‐	‐	‐	140	110	62	78	98	63	39
	Pig 2	*	22	*	*	*	*	‐	‐	‐	‐	135	132	78	95	99	54	39.9
	Pig 3	‐	20	450	48	15	2:0	7.41	241	48	30.4	114	108	67	80	98	47	37.3
	Pig 4	28	28	250	45	15	1:1.3	7.42	261	48	31.1	104	93	54	68	99	52	38.7
14	Pig 3	20	18	475	36	5	‐	7.55	243	33	28.9	96	126	81	100	98	36	39.1
	Pig 4	25	17	468	50	5	1:2	7.51	267	40	31.9	68	101	54	74	99	43	37.6
16	Pig 3	23	18	475	47	5	1:1.48	7.47	237	43	31.3	82	127	83	102	98	43	38.1
	Pig 4	25	17	470	51	5	1:2	7.48	249	46	33.5	76	105	58	77	99	46	37.8
18	Pig 3	25	18	400	47	10	1:1.48	7.44	235	49	33.3	80	121	78	96	98	48	38
	Pig 4	28	17	471	51	10	1:2	7.47	247	50	36.4	81	101	54	73	99	56	38.3
20	Pig 3	26	18	400	46	10	1:1.48	7.42	233	53	34.4	80	119	71	89	98	54	38.8
	Pig 4	28	20	470	46	10	1:2	7.46	246	48	31.2	76	95	46	65	99	53	38.2
22	Pig 3	28	22	350	54	15	‐	7.4	227	55	34.1	74	124	89	97	97	53	38.6
	Pig 4	30	24	471	46	15	1:1.8	7.43	224	52	34.5	75	92	45	62	99	58	38.2
24	Pig 3	26	22	350	53	15	‐	7.41	247	53	33.6	75	103	68	78	97	51	38.4
	Pig 4	40	24	474	46	15	1:1.4	7.33	230	52	30.8	77	90	46	61	99	65	38.6

Abbreviations: DAP, diastolic arterial pressure; ETCO_2_, end tidal carbon dioxide; FiO_2_, fraction of inspired oxygen; HR, heart rate; Hour, time from first blood gas sampling; I,E ratio, inspiratory to expiratory time ratio; MAP, mean arterial pressure; PEEP, peak end expiratory pressure; PIP, peak inspiratory pressures; PO_2_, partial pressure of oxygen in arterial blood; PCO_2_, partial pressure of carbon dioxide in arterial blood; RR, respiratory rate; SAP, systolic arterial pressure; SPO_2_, TV, tidal volume; oxygen saturation via pulse oximetry; Temp, temperature.

‐: Data not recorded.

*Novel ventilator prototype failure.

## 3 DISCUSSION

The median alfaxalone infusion rate in our study was comparable to other studies in pigs in which alfaxalone was infused alone (4.8 mg/kg/h) (Bigby et al., 2017). Similar alfaxalone infusion rates (5 mg/kg/h) in combination with ketamine 5 mg/kg/h and dexmedetomidine 4 μg/kg/h were reported in another study following premedication with ketamine and midazolam (Lervik et al., 2020). Both studies maintained anaesthesia for only 60 min, and it is likely that the pre‐anaesthetic drugs influenced drug infusion rates during the short anaesthetic period. For example, Bigby et al. (2017) observed that in some animals (3/9), endotracheal intubation was possible with the premedication only and no additional induction drug was required.

The protocol used in the present study was suitable for immobilising the pigs for mechanical ventilation but would not be suitable for noxious interventions. This is evidenced by most animals requiring additional alfaxalone or methadone when stimulated to position for radiographs. Interestingly, similar findings were reported during the previously described short duration alfaxalone and alfaxalone‐ketamine‐dexmedetomidine infusions with approximately 14%–35% of animals responding to epidural placement or dewclaw clamping, respectively (Bigby et al., 2017; Lervik et al., 2020). Alfaxalone is not an analgesic, and while information on dexmedetomidine in pigs is limited, it has been reported to provide significant analgesia when infused at 4 mcg/kg/h in combination with ketamine and propofol (Lervik et al., 2020). It is possible that increasing the dexmedetomidine infusion rate and perhaps the addition of an opioid to the infusion regime may have provided more consistent anti‐nociception and a more balanced anaesthetic and analgesic technique.

Cardiovascular parameters monitored were within acceptable ranges for anaesthetised pigs, and mean arterial blood pressure did not fall below 60 mmHg in any animal despite periods of significant positive end expiratory pressure of up to 15 cmH_2_O and peak inspiratory pressures (PIP) often in excess of 25 cm H_2_O (see Table 2 for details). This is in agreement with other studies investigating the cardiovascular effects of alfaxalone‐based anaesthetic protocols in pigs (Duval et al., 2018; Ruane‐O'Hora et al., 2011).

Previously, reports of alfaxalone (without preservative) infused alone or as part of a balanced anaesthetic technique in pigs have reported maintenance of anaesthesia for up to 60 minutes duration (Bigby et al., 2017; Duval et al., 2018; Lervik et al., 2020). To the authors knowledge, this is the first report of the preserved formulation of alfaxalone infused for 12–24 h to maintain anaesthesia in pigs. The formulation of alfaxalone used in the present study contains preservatives (ethanol, chlorocresol and benzethonium chloride); we are unable to rule out any possible toxicity or unforeseen effects these preservatives might have caused.

In conclusion, this study demonstrated that a combination of alfaxalone and dexmedetomidine is suitable to maintain long duration TIVA in pigs anaesthetised for controlled mechanical ventilation.

## AUTHOR CONTRIBUTIONS

*Formal analysis and writing—original draft*: Irving Kat. *Resources and writing—review and editing*: Jayesh Dhanani. *Investigation and writing—review and editing*: Grant Whitten. *Investigation and writing—review and editing*: Nicholas Cowling. *Conceptualisation, investigation, methodology, writing—original draft, and writing—review and editing*: Wendy Goodwin.

## CONFLICT OF INTEREST

The authors declare no conflict of interest.

## FUNDING INFORMATION

None.

### PEER REVIEW

The peer review history for this article is available at https://publons.com/publon/10.1002/vms3.953.

## ETHICS STATEMENT

The authors confirm that the ethical policies of the journal, as noted on the journal's author guidelines page, have been adhered to and the appropriate ethical review committee approval has been received. The study protocol was approved by the University of Queensland Animal Ethics Committee in accordance with the Australian code for the care and use of animals for scientific purposes.

## Data Availability

The data that support the findings of this study are available from the corresponding author upon reasonable request.
